# The NR2F2-HAND2 signaling axis regulates progesterone actions in the uterus at early pregnancy

**DOI:** 10.3389/fendo.2023.1229033

**Published:** 2023-08-18

**Authors:** Yeongseok Oh, Elvis Quiroz, Tianyuan Wang, Yassmin Medina-Laver, Skylar Montague Redecke, Francisco Dominguez, John P. Lydon, Francesco J. DeMayo, San-Pin Wu

**Affiliations:** ^1^ Reproductive and Development Biology Laboratory, National Institute of Environmental Health Sciences, Research Triangle Park, NC, United States; ^2^ Department of Life Science and Research Institute for Natural Sciences, Hanyang University, Seoul, Republic of Korea; ^3^ Center for Reproductive Biology, School of Molecular Biosciences, College of Veterinary Medicine, Washington State University, Pullman, WA, United States; ^4^ Biostatistics & Computational Biology Branch, National Institute of Environmental Health Sciences, Research Triangle Park, NC, United States; ^5^ IVIRMA Global Research Alliance, IVI Foundation, Instituto de Investigación Sanitaria La Fe (IIS La Fe), Valencia, Spain; ^6^ Department of Molecular and Cellular Biology, Baylor College of Medicine, Houston, TX, United States

**Keywords:** progesterone signaling, NR2F2 (COUP-TFII), *Hand2* transcription factor, endometrial stroma, preimplantation, pregnancy, CRISPR activation (CRISPRa), enhancer

## Abstract

Endometrial function is dependent on a tight crosstalk between the epithelial and stromal cells of the endometrium. This communication is critical to ensure a fertile uterus and relies on progesterone and estrogen signaling to prepare a receptive uterus for embryo implantation in early pregnancy. One of the key mediators of this crosstalk is the orphan nuclear receptor NR2F2, which regulates uterine epithelial receptivity and stromal cell differentiation. In order to determine the molecular mechanism regulated by NR2F2, RNAseq analysis was conducted on the uterus of *Pgr^Cre^;Nr2f2^f/f^* mice at Day 3.5 of pregnancy. This transcriptomic analysis demonstrated *Nr2f2* ablation in *Pgr*-expressing cells leads to a reduction of *Hand2* expression, increased levels of *Hand2* downstream effectors *Fgf1* and *Fgf18*, and a transcriptome manifesting suppressed progesterone signaling with an altered immune baseline. ChIPseq analysis conducted on the Day 3.5 pregnant mouse uterus for NR2F2 demonstrated the majority of NR2F2 occupies genomic regions that have H3K27ac and H3K4me1 histone modifications, including the loci of major uterine transcription regulators *Hand2*, *Egr1*, and *Zbtb16*. Furthermore, functional analysis of an NR2F2 occupying site that is conserved between human and mouse was capable to enhance endogenous *HAND2* mRNA expression with the CRISPR activator in human endometrial stroma cells. These data establish the NR2F2 dependent regulation of *Hand2* in the stroma and identify a cis-acting element for this action. In summary, our findings reveal a role of the NR2F2-HAND2 regulatory axis that determines the uterine transcriptomic pattern in preparation for the endometrial receptivity.

## Introduction

The establishment and maintenance of a successful pregnancy depends on the signaling of sex steroid hormones estrogen and progesterone, which regulate critical pathways involved in embryo implantation and other gestational processes. (1, 2). Whereas estrogen acting through the estrogen receptor alpha (ESR1) induces the proliferation of uterine epithelial cells, progesterone acting through the progesterone receptor (PGR) orchestrates the crosstalk between epithelial and stromal compartments of the endometrium to counteract the estrogen-induced epithelial proliferation (2). Disruption of estrogen and progesterone signaling leads to female reproductive pathology, such as preeclampsia, gestational diabetes, preterm birth, and recurrent spontaneous abortions, leading towards an unsuccessful pregnancy (3–5).

Chicken ovalbumin upstream promoter transcription factor II (COUP-TFII, NR2F2) is an orphan nuclear receptor with transcription factor activities involved in diverse biological processes including angiogenesis, adipogenesis, neuronal development, organogenesis, reproduction during embryonic development, tissue remodeling, and disease progression (6–9). NR2F2 is essential for both male and female fertility (10, 11). In the human reproductive system, NR2F2 regulates expression of multiple genes for inflammatory baseline determination in endometrial stroma cells (12) and plays a role in pathogenesis of endometriosis (13, 14). In the mouse uterus, NR2F2 is expressed in both the stromal and the myometrial compartment (10). Ablation of NR2F2 in PGR-expressing cells results in female infertility due to embryo implantation failure (15). NR2F2 in the stromal cells promotes BMP2 expression for decidualization and mediates endometrial PGR signaling to oppose epithelial estrogen activities and inhibit epithelial proliferation in preparation of a receptive endometrium for subsequent embryo implantation (15, 16). Additionally, NR2F2 is required for stromal PGR protein expression in an ovariectomized mouse model that receives exogenous progesterone and estrogen treatment to mimic pregnancy at gestation day 3.5 (15). Despite of the pivotal role of NR2F2 in epithelial-stromal communication, the mechanism by which NR2F2 suppresses estrogen-mediated epithelial proliferation remains unclear.

Since the basic helix-loop-helix transcription factor (HAND2) is downstream of endometrial progesterone signaling and acts through fibroblast growth factors (FGFs) to suppress epithelial proliferation, endometrial interaction between NR2F2 and HAND2 has been proposed in mediating progesterone dependent epithelial-stromal crosstalk for the control of epithelial proliferation at early pregnancy (2, 17). Like NR2F2, HAND2 also regulates expression of genes that control the immune baseline in endometrial stroma and decidual cells (18–20). The present study examines the impact of *Nr2f2* loss in *Pgr*-expressing cells on the uterine transcriptome and on *Hand2* gene expression in a natural pregnancy context, followed by comparative transcriptomic analyses, NR2F2 ChIP-seq and the CRISPR activation assay, to provide insight on the mechanisms by which NR2F2 directs downstream gene expression to modulate the inter-compartmental communication in the uterine endometrium.

## Materials and methods

### Experimental animals

*Nr2f2* ^flox/flox^ (*Nr2f2* ^f/f^) and *Pgr*-Cre knockin (*Pgr* ^Cre/+^) mice have been generated previously (10, 21). Conditional knockout mice of *Nr2f2*, *Pgr* ^Cre/+^; *Nr2f2* ^f/f^ (*Nr2f2* ^d/d^) were generated by crossing *Pgr* ^Cre/+^ mice with *Nr2f2* ^f/f^ mice. *Nr2f2* ^f/f^ mice were used as wild type. Wild type and *Nr2f2* ^d/d^ female mice at 2 - 3 months of age were mated with vasectomized male mice. The morning of a vaginal plug was designated as gestational day (GD) 0.5. The uterine tissues were obtained from the mice at GD 3.5. For ChIP assay experiment, normal C57BL/6 female mice at 2 - 3 months of age were mated with vasectomized male mice, and then the uterine tissues were obtained at GD 3.5. All animal experiments were performed in accordance with National Institute of Environmental Health Sciences (NIEHS) Animal Care and Use Committee guidelines and in compliance with NIEHS-approved animal protocol (ASP 2015-0012 and 2015-0023).

### Quantitative RT-PCR

Total RNA was isolated from uterine tissues using TRIzol reagent (Thermo Fisher Scientific), and then RT was performed using MMLV reverse transcriptase (Thermo Fisher Scientific). Real-time PCR was performed using SsoAdvanced Universal Probes Supermix (Bio-Rad Laboratories) and Taqman probes (Thermo Fisher Scientific) in a CFX Connect Real-time PCR Detection System (Bio-Rad Laboratories). Real-time PCR was performed using the following protocol: 5 min at 95°C, 45 cycles of denaturation (10 sec at 95°C) and annealing/extension (30 sec at 60°C), and a final step of melting curve analysis. CFX Manager software (version 3.0, Bio-Rad Laboratories) was used to collect the PCR data. As an internal control, 18S rRNA endogenous control (Applied Biosystems) was used. The relative level of mRNA was calculated using the 2^-ΔΔCt^ method. The primers are listed in **Table S1**.

### RNA-seq

Total RNA was isolated from uterine tissues using TRIzol reagent (Thermo Fisher Scientific), and then the RNA quality was assessed using the Agilent 2100 Bioanalyzer (Agilent Technologies). RNA-seq was performed by the Epigenomics and DNA Sequencing Core Laboratory at NIEHS. RNA libraries were generated using TruSeq RNA Library Prep kit v2 (Illumina), and then sequenced using NextSeq 500 system (Illumina).

The raw reads (76-bp, paired-end) were initially processed by applying a filter with an average quality score of 20. Adaptor sequences were subsequently removed from reads using cutadapt (v1.12). The processed reads were then aligned to the mouse reference genome (mm10) using the STAR aligner (v2.5.2b). The gene expression levels in each sample were determined by counting the total number of paired-end reads mapped to each gene using the featureCounts (v1.5.0-p1) function within the Subread program. Differential gene expression analysis was conducted using the DESeq2 R package (v1.12.4) with a significance threshold of absolute fold change ≥ 1.4 and a p-value < 0.05 (22).

### Immunohistochemistry

Uterine tissues were fixed in 4% paraformaldehyde and then embedded in paraffin. Sections (5 µm) were mounted on glass slides, deparaffinized, and rehydrated. Antigen retrieval was performed using Antigen Unmasking Solution (H-3300, Vector Laboratories, Burlingame, CA) according to manufacturer’s instructions. The slides were incubated in 3% H_2_O_2_ in methanol to block endogenous peroxidase activity. Then, the samples were blocked using 5% normal goat (for rabbit primary antibodies) or rabbit serum (for goat primary antibody). The slides were incubated in primary antibodies overnight at 4°C. After washing in PBS, the slides were incubated in peroxidase-labeled goat anti-rabbit IgG (1:200; BA-1000, Vector Laboratories) or rabbit anti-goat IgG (1:200; BA-5000, Vector Laboratories) for 30 min. The ABC reagent (PK-6100, Vector Laboratories) was applied to slides according to manufacturer’s instructions. The signal was developed using a 3,3’-diaminobenzidine (DAB) substrate kit (SK-4105, Vector Laboratories), the nuclei were stained using hematoxylin, and the slides were permanently mounted. Information of primary antibodies is provided in **Table S2**.

### ChIP-seq and ChIP-qPCR

Uterine tissues were minced and fixed in 1% formaldehyde for 10 min and quenched with 0.125 M glycine. The samples were washed with cold PBS containing protease inhibitors, resuspended in homogenization buffer [50 mM Tris-HCl (pH 7.5), 1 mM EDTA (pH 8.0), 1% NP-40 (IGEPAL), 0.25% deoxycholic acid, and protease inhibitors], and then homogenized with a Polytron (Kinematica). The samples were pelleted by centrifugation, resuspended in membrane lysis buffer [5 mM PIPES (pH 8.0), 85 mM KCl, 0.5% NP-40 (IGEPAL), and protease inhibitors], and incubated on ice for 5 min. The samples were pelleted by centrifugation, resuspended in nuclear lysis buffer [50 mM Tris-HCl (pH 8.0), 10 mM EDTA (pH 8.0), 1% SDS and protease inhibitors], and incubated on ice for 10 min. Lysates were sonicated, and the DNA was sheared into an average length of 300-400 bp. Chromatin was diluted 1:9 with dilution buffer [50 mM Tric-HCl (pH 8.0), 2 mM EDTA (pH 8.0), 300 mM NaCl, 1% NP-40 (IGEPAL), 0.5% sodium deoxycholate, 0.1% SDS, and protease inhibitors] and then precleared with Dynabeads Protein A (Invitrogen, Carlsbad, CA). Chromatin immunoprecipitation (ChIP) was performed using the antibodies for NR2F2 (61213, Active Motif) and PGR (8757, Cell Signaling Technology). After incubation at 4°C overnight, Dynabeads Protein A was used to isolate the immune complexes. Complexes were washed in wash buffer [20 mM Tris- HCl (pH 8.0), 2 mM EDTA (pH 8.0), 300 mM NaCl, 0.1% SDS, 1% Triton X-100, and protease inhibitors] and eluted using elution buffer [100 mM NaHCO_3_ and 1% SDS]. Cross-links were reversed in 200 mM NaCl by incubation overnight at 65°C, and then the samples were treated with RNase and Proteinase K. ChIP DNA was purified by phenol-chloroform extraction and isopropanol precipitation. The purified DNAs were subjected to sequencing or quantitative PCR. The ChIP DNA libraries were generated using NEXTflex Rapid DNA-Seq kit (Perkin Elmer), and then sequenced using the NextSeq 500 system (Illumina). H3K4me1 ChIP was performed by Active Motif, Inc. Whole uterine tissue of mice at GD 3.5 were flash frozen and sent to Active Motif. The tissue was fixed and then promptly sheared into small fragments before immunoprecipitation with the H3K4me1 antibody (cat # 39297, Active Motif, Carlsbad, CA). DNA was purified and amplified to generate a library and using the NextSeq 500 system (Illumina).

The raw reads (75-bp, single-end) were subjected to filtering, retaining only reads with average quality scores greater than 20. Subsequently, the reads were aligned to the mouse reference genome (mm10) using Bowtie (v1.1.2) (23), requiring unique mapping and allowing up to 2 mismatches per read (-m 1 -v 2). Duplicated reads with identical sequences were removed using Picard tools. To visualize the read coverage, BigWig files were generated from the bedgraph files of each sample using bedGraphToBigWig. These bigWig files were displayed as custom tracks on the UCSC genome browser. The initial peaks within each dataset were identified using MACS2, applying a cutoff of adjusted p-value < 0.0001 (24). To obtain a comprehensive set of peaks across all ChIP-seq datasets, the peak intervals from each dataset were merged using BedTools. The gene associated with each peak was predicted by searching the transcription start site (TSS) of nearby genes within a 50Kb range using HOMER (25).

For ChIP quantitative PCR (ChIP-qPCR), primers were designed to amplify regions of NR2F2 occupancy identified through ChIP-seq. ChIP-qPCR was performed in duplicate on specific genomic regions using SsoAdvanced Universal SYBR Green Supermix (Bio-Rad Laboratories). As a negative control, the Mouse Negative Control Primer Set 1 (Active Motif) which amplifies a fragment in a gene desert on mouse chromosome 6 was used. The resulting signals were normalized to input DNA and presented as percentage to input. The primer sequences are listed in **Table S3**.

### CRISPR activation assay

The following experiments were conducted in immortalized T-HESC cells (American Type Culture Collection, Cat. #4003TM), a model cell line of the human endometrial stroma cells (26). T-HESC cells were transduced with lentivirus that contained IGI-P0492 pHR-dCas9-NLS-VPR-mCherry (Research Resource Identifier: Addgene_102245) and guide RNA (gRNA)-green fluorescent protein (GFP)-expressing vectors. IGI-P0492 pHR-dCas9-NLS-VPR-mCherry was a gift from Jacob Corn (Addgene plasmid # 102245; http://n2t.net/addgene:102245; RRID : Addgene_102245). gRNA-GFP-expressing vectors were synthesized with the respective guide sequences listed in **Table S4** by the VectorBuilder (Chicago, IL). The non-targeting controls were included on each experimental batch by following the same procedure.

The cells were infected by the mentioned lentivirus with a MOI of 4 for the IGI-P0492 pHR-dCas9-NLS-VPR-mCherry lentivirus and with a MOI of 4 for the gRNA-GFP-expressing vectors, including the non-targeting control. After infection for 72h at 37°C, the cells were sorted selecting cells positive for both GFP and mCherry fluorescence. Then, cells were harvested, RNA was extracted, complementary DNA (cDNA) was synthetized and qRT-PCR was applied to assess the effect of infection. All the RT-PCR results were normalized to the expression levels of the internal control 18S rRNA. The 18S rRNA probe is from ThermoFisher Scientific (4319413E, ThermoFisher Scientific, Waltham, MA) and primers for *HAND2* are listed in **Table S1**.

Cells were maintained in the Dulbecco’s modified Eagle medium (DMEM)/F12 medium (Thermo Fisher 11330032) with 10% fetal bovine serum (Thermo Fisher, #10437028) and 1× antibiotic–antimycotic (Thermo Fisher 15240062).

### Data analysis

Statistical significance was analyzed using GraphPad Prism 8 (GraphPad Software). The differentially expressed genes generated by RNA-seq were analyzed using Ingenuity Pathway Analysis software (IPA, Qiagen Bioinformatics). The gene signature assay was performed on the Gene Set Enrichment Analysis (GSEA) (27). The correlation study between *Nr2f2* ^d/d^ transcriptome profile and public data sets was performed using Correlation Engine software (BaseSpace, Illumina). The ChIP-seq data were analyzed using UCSC Genome Browser (https://genome.ucsc.edu/) (28) and Cistrome software (http://cistrome.org/ap/) (29). The CRISPRa validation statistic was analyzed using Friedman’s test. Data are presented as the mean ± SEM.

## Results

### NR2F2 loss in mouse uterus results in unopposed estrogen activities and a change of immune gene expression

**Figure 1:**
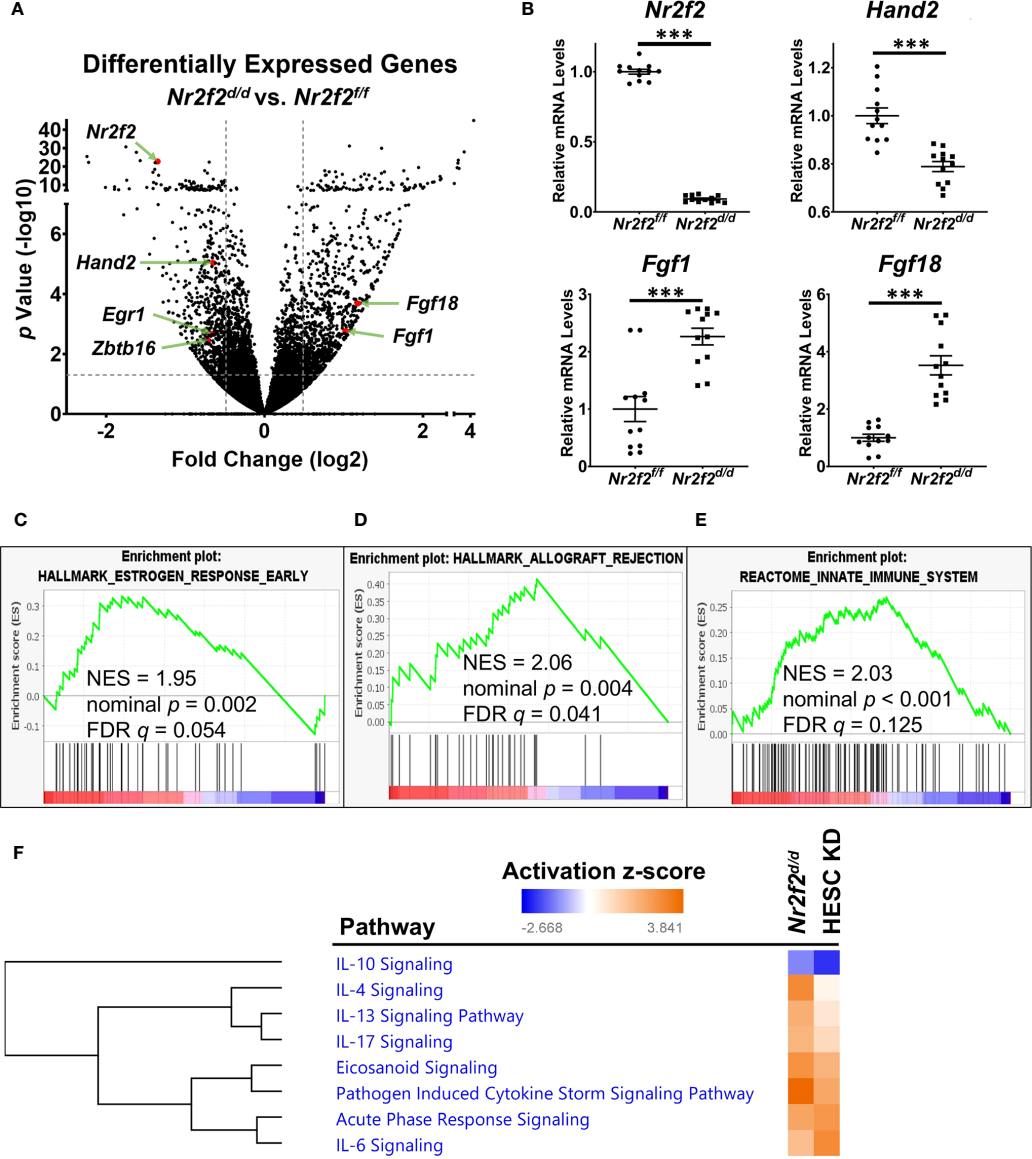
NR2F2 downstream genes and associated pathways in the GD3.5 mouse uterus. **(A)** A volcano plot of the differentially expressed genes between mutant and control uteri. The horizontal dotted line marks the unadjusted p value at 0.05. Vertical dotted lines label the fold change cutoff at 1.4 and -1.4. Selected genes of interest are marked in red with gene symbols displayed. N=4 for each group. **(B)** RT-qPCR validation of the relative RNA abundance of denoted genes. Each group has datapoints of 6 biological replicates with technical PCR duplicates. ***, *p*< 0.001 by two-tailed Mann Whitney test. Error bars denote the standard error of the mean. **(C–E)** Enrichment of NR2F2 downstream genes in molecular pathways by GSEA. NES, normalized enrichment score; FDR, false discovery rate. **(F)** Selected inflammatory pathways that have over-representation of NR2F2 downstream genes in mouse and human, identified by the IPA Canonical Pathway Analysis. Pathways are grouped by hierarchical clustering of activation z-scores. HESC KD, *NR2F2* knockdown in human endometrial stroma cells (NCBI GEO accession GSE47052).

Gestation day 3.5 (GD3.5) uterine tissues from *Nr2f2^f/f^* (*Nr2f2^flox/flox^*) and *Nr2f2^d/d^* (*Pgr^Cre/+^; Nr2f2^flox/flox^*) mice were subject to the RNA-seq assay to examine the impact of *Nr2f2* ablation on the transcriptomic profile. A total of 1533 differentially expressed genes (DEGs) were identified (**Figure 1A**, **Dataset S1**). A reduction of *Nr2f2* mRNA levels in the *Nr2f2^d/d^* uterus confirmed the ablation of *the Nr2f2* gene and validated the model (**Figures 1A, B**, **Dataset S1**). The mRNA abundance of major uterine transcription regulators for embryo implantation, including *Hand2*, *Egr1*, and *Zbtb16* among others (17, 30, 31), is lower in *Nr2f2^d/d^* compared with control mice (**Figures 1A, B**, **Dataset S1**), which indicates that NR2F2 is upstream of these transcription factors in the genetic network. Notably, *Nr2f2* ablation also leads to an increase in mRNA abundance of HAND2 downstream targets *Fgf1* and *Fgf18* (**Figure 1A, B**, **Dataset S1**). Moreover, the *Nr2f2^d/d^* uterine transcriptome manifests an estrogen hypersensitive phenotype, as shown by the positive correlation with expression profiles of estrogen responsive genes in the Gene Set Enrichment Analysis (GSEA) (**Figure 1C**) and in the Ingenuity Pathway Analysis (IPA) (beta-estradiol in IPA upstream regulators of **Dataset S1**). Since stromal *Hand2* suppresses endometrial estrogen signaling at GD3.5 (17), the impact of *Nr2f2* loss on the *Hand2*-*Fgf* pathway supports NR2F2 as an upstream regulator of *Hand2* in modulating epithelial-stromal communication in the endometrium for embryo receptivity.

In addition to estrogen hypersensitivity, the *Nr2f2^d/d^* transcriptome manifests a change in the immune baseline as indicated by the positive correlation with the GSEA curated datasets of “allograft rejection” and “innate immune system” (**Figures 1D, E**). This *in vivo* observation is consistent with the enrichment of inflammation-associated genes in the NR2F2 regulated transcriptome of the human endometrial stroma cells (HESC) (12). Based on NR2F2 downstream target gene lists, the Ingenuity Pathway Analysis (IPA) canonical pathway assay further reveals the impact of NR2F2 loss on immune and inflammation associated signaling pathways in both mouse and human cell models, involving not only both pro- and anti-inflammatory cytokines such as IL6, IL17, IL4, IL10, and IL13, but also mechanisms for pathological response such as “pathogen induced cytokine storm signaling pathway”, “acute phase response signaling”, and “ eicosanoid signaling” (**Figure 1F**, **Dataset S2**). These findings suggest that NR2F2 loss creates a gene expression pattern permissive for immune challenges and that this phenotype is conserved between species.

Notably, expression of many extracellular matrix and cell-matrix interaction genes are altered in response to NR2F2 loss as captured under “wound healing signaling pathway” and “integrin signaling” (IPA canonical pathways of **Dataset S1**), which implicates a role of NR2F2 in regulation of the uterine matrix homeostasis that is important for sustaining a normal pregnancy (32). These observations provide insights in a molecular context for the embryo implantation failure phenotype in mutant mice (15). In summary, this RNAseq data revealed multiple mechanisms utilized by NR2F2 to sustain normal uterine function at early pregnancy.

### Positively correlated gene signatures between NR2F2 and major uterine regulators

**Figure 2:**
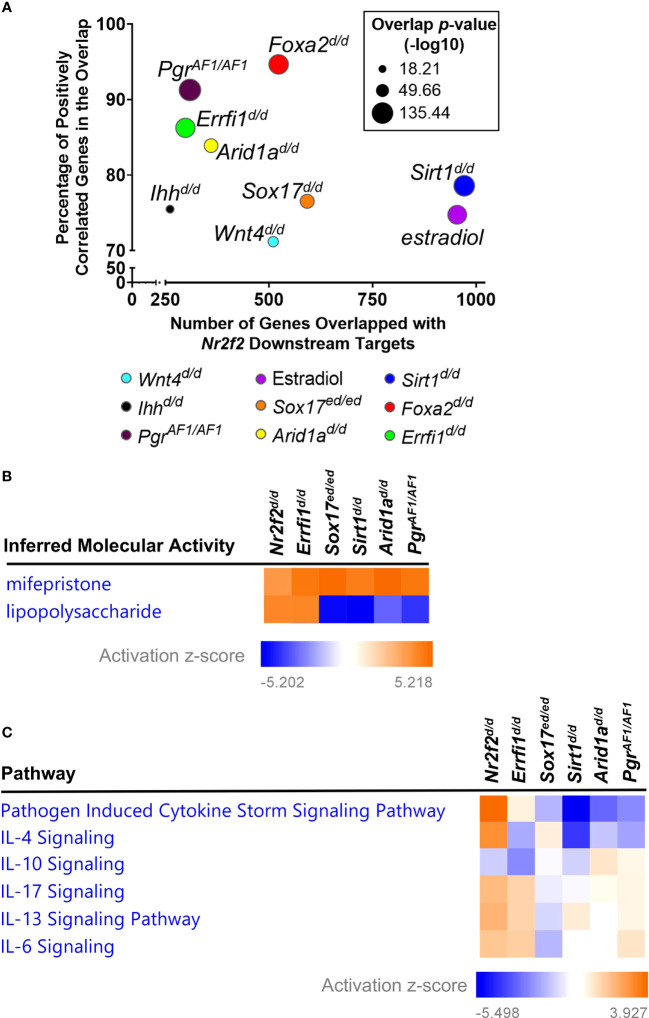
Gene signature comparison between *Nr2f2* and major uterine regulators. **(A)** Common downstream genes by the Illumnia BaseSpace Correlation engine. **(B, C)** Comparative analysis of gene signatures on inferred molecular activities by the IPA upstream regulator assay **(B)** and on enrichment of impacted genes in curated signaling pathways by the IPA canonical pathway assay **(C)**.

Next we compared the transcriptomic changes between NR2F2 ablation and other major uterine regulator perturbations that are crucial for early pregnancy. Subsets of the 1533 DEGs from NR2F2 ablation are present in DEGs from mutations of PGR Activation function 1 domain (*Pgr^AF1/AF1^*, 309 genes) (33), *Ihh* (*Ihh^d/d^*, 261 genes) (34), *Sox17* (*Sox17^d/d^*, 592 genes) (35), *Foxa2* (*Foxa2^d/d^*, 523 genes) (36), *Wnt4* (*Wnt4^d/d^*, 510 genes) (37), *Errfi1* (*Errfi1^d/d^*, 298 genes) (38), *Arid1a* (*Arid1a^d/d^*, 360 genes) (39), and *Sirt1* (*Sirt1^d/d^*, 971 genes) (40) (**Figures 2A**, **S1**). Most overlapped genes shared the same direction of gene expression alterations in response to perturbations (91% with *Pgr^AF1/AF1^*, 75% with *Ihh^d/d^*, 77% with *Sox17^d/d^*, 94% with *Foxa2^d/d^*, 71% with *Wnt4^d/d^*, 86% with *Errfi1^d/d^*, 84% with *Arid1a^d/d^*, and 79% with *Sirt1^d/d^*), indicating a positive correlation on gene signatures between NR2F2 and these major uterine regulators (**Figures 2A**, **S1**). These findings provide transcriptomic evidence to support interactions between NR2F2 and other uterine regulators for gene expression programs at early pregnancy.

Consistent with the previously observed estrogen hyperactivity (15, 16), the *Nr2f2^d/d^* transcriptome is positively correlated with the uterine estradiol gene signature, with 75% of overlapping genes sharing the same direction of changes (**Figures 2A**, **S1**). Moreover, the result of the IPA upstream regulator assay reveals an increase of inferred mifepristone activities in the *Nr2f2^d/d^* transcriptome (**Figure 2B**), suggesting a loss of progesterone signaling activities. This genome-wide evidence supports the phenotype of unopposed estrogen activities in the context of progesterone signaling loss, in addition to the marker-gene-based findings in **Figure 1**. In addition, this progesterone signaling loss phenotype is seen in the GD3.5 uterine transcriptome of *Pgr^AF1/AF1^*, *Sox17^d/d^*, *Errfi1^d/d^*, *Arid1a^d/d^*, and *Sirt1^d/d^* mice that manifest hyperactive estrogen activities (**Figure 2B**) (33, 35, 38–40), indicating a common theme among these transcription regulators in modulating progesterone signaling activities. On the other hand, these uterine regulators also exhibit a divergent change of the immune baseline despite their shared loss of progesterone signaling activities, as demonstrated by the inferred lipopolysaccharide activities, a surrogate for the inflammation status, manifesting an increase in *Nr2f2^d/d^* and *Errfi1^d/d^* and a reduction in *Pgr^AF1/AF1^*, *Sox17^d/d^*, *Arid1a^d/d^*, and *Sirt1^d/d^* uteri (**Figure 2B**). The difference on the change of the immune baseline is further reflected by the diverse enrichment pattern of cytokine signaling pathways that are under the control of these regulators (**Figure 2C**). These findings demonstrate the common and distinct biological processes between NR2F2 and other major uterine regulators in the uterus at early pregnancy.

### Reduced HAND2 protein abundance in the *Nr2f2* deficient uterine stroma

**Figure 3:**
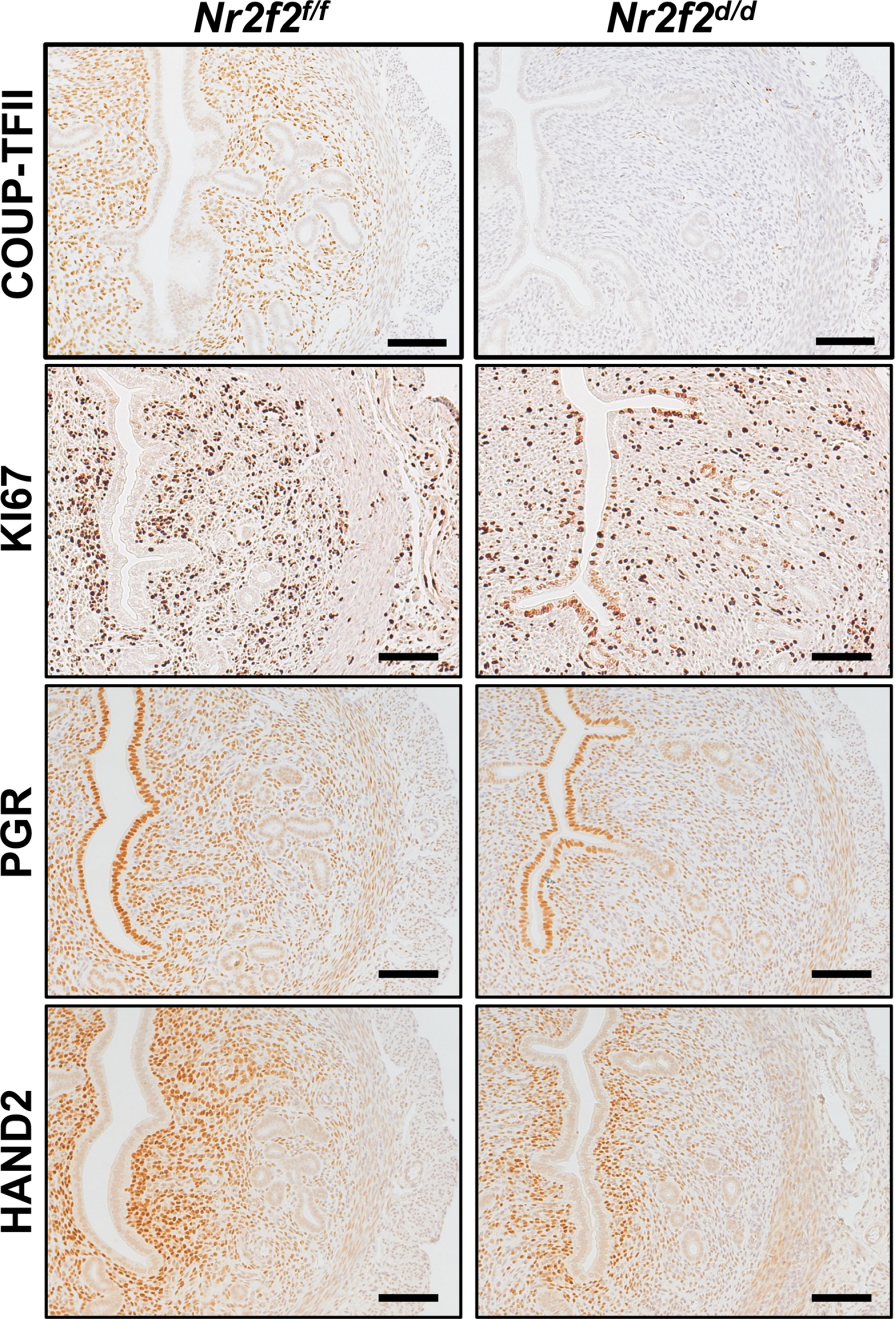
Immunostaining of denoted protein on the GD3.5 uterus. Four biological replicates on each genotype. Scale bar = 100 um.

Immunostaining results reveal multiple KI67-positive cells in the luminal epithelium and a reduction of PGR expression levels in the stroma of the *Nr2f2^d/d^* uterus at GD3.5, in contrast to the negative KI67 signal and strong PGR expression in the corresponding compartments of the control uterus (**Figure 3**). This observation indicates the presence of unopposed estrogen signaling activities in the mutant uterus, which is consistent with the RNAseq findings (**Figure 1C**) and previous reports (15, 16). In the mutant stroma, the HAND2 protein abundance is lower compared with the control (**Figure 3**), in line with the changes in the uterine *Hand2* mRNA levels and reflecting the de-repression of HAND2’s downstream targets *Fgf1* and *Fgf18* (**Figure 1B**). Collectively, the immunohistochemistry findings further support the transcriptomic results on the estrogen hypersensitive phenotype and the impact on stromal HAND2 expression.

### Tissue specific NR2F2 genome occupancy pattern in the uterus

**Figure 4:**
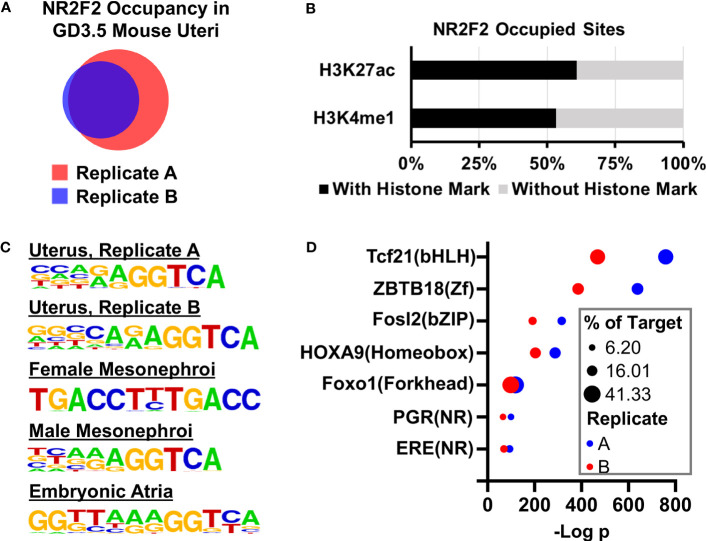
NR2F2 genome occupancy in the GD3.5 uterus by ChIP-seq. **(A)** Comparision of NR2F2 occupied intervals in two pools of wild type uterine tissues. **(B)** Fractions of NR2F2 occupied sites with and without enhancer histone marks. **(C)** NR2F2 binding motifs in various mouse tissues predicted by the HOMER *de novo* motif analysis based on NR2F2 ChIP-seq data. **(D)** Selected known motifs of uterine regualtor families that are enriched in NR2F2 occupied sites in the uterus.

Given the transcription factor feature of NR2F2, the NR2F2 genome occupying profile was mapped by the ChIP-seq assay in the GD3.5 uterus using two pools (Replicates A and B) of wild type mouse uteri. Replicates A and B have 14150 and 8285 NR2F2 occupied genomic intervals, respectively, and share 7425 common intervals (**Figure 4A**, **Dataset S3**). Over 60% and 53% of the 7425 NR2F2 occupied intervals are located in the H3K27ac (41) and H3k4me1 positive histone marks loci, respectively (**Figure 4B**). This observation suggests that the majority of genome-binding NR2F2 is located in active, poised enhancers, or both. *De novo* motif enrichment analysis shows an overrepresentation of the (A/G)GGTCA sequence in both replicates of the GD3.5 uterus (**Figure 4C**). Such an enrichment is also seen in NR2F2 occupying sites that were mapped previously in female and male mesonephori (9) and in the embryonic atria (42). Results of this unbiased search of the *in vivo* NR2F2-binding motif for mice are consistent with previous *in vitro* findings (43). In addition, this sequence is also preserved in NR2F2 occupying sites in human cells (12, 44). Moreover, both replicates show overrepresentations of motifs of major uterine regulators including PGR, ESR1, and AP-1 among others (**Figure 4D**, **Dataset S4**). Notably, motifs of two major uterine regulators PGR and FOXL2 are overrepresented only in the uterus in a survey of the NR2F2 cistrome across various tissues (**Dataset S4**), while motifs for CTCF/CTCFL and TBX20 are only enriched in embryonic mesonephroi and atria, respectively (**Dataset S4**). These results suggest a potential tissue-specific cistome microenvironment that permits interactions of NR2F2 with a unique group of transcription regulators to modulate gene expression. In summary, the similar findings between these two replicates together support the validity of NR2F2 ChIP-seq results in the uterus. These observations also provide a comprehensive map of the uterine NR2F2 cistrome and candidate interacting factors on genomic regulation at GD3.5.

### *Hand2* as a direct downstream target gene of NR2F2

**Figure 5:**
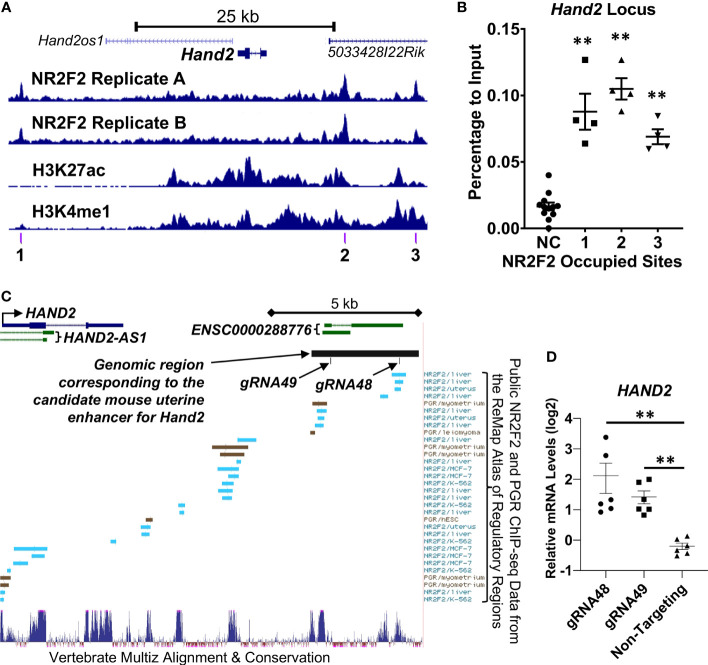
NR2F2 occupany and enhancer histone mark patterns at the *Hand2* locus in the GD3.5 uterus. **(A)** Trackview of the *Hand2* locus **(B)** ChIP-qPCR of the GD3.5 uterus on NR2F2 occupying regions denoted in **(A)**. NC, Negative control site. **(C, D)** Functional assessment of a candidate uterine enhancer for HAND2 gene regulation in T-HESC. **(C)** Track view of the UCSC Genome Browser at the human *HAND2* locus in the hg38 genome assembly with annotations of the gRNAs for CRISPRa and the genomic region corresponding to a NR2F2 occupied mouse uterine enhancer (chr8:57331591-57335031 in the mm10 genome assembly). **(D)** Relative HAND2 mRNA abundance by RT-qPCR after targeting the candidate enhancer by CRISPRa. Each group includes datapoints of biological triplicates with technical PCR duplicates. **, *p* < 0.01 by the two-tailed Mann Whitney test. Error bars denote the standard error of the mean.

Both NR2F2 uterine ChIP-seq replicates show NR2F2 occupancy at the *Hand2* locus (**Figure 5A**). ChIP-qPCR validated the NR2F2-binding events in all three NR2F2-binding intervals surrounding the *Hand2* gene body within a 52-kilobase stretch of genomic region (**Figures 5A, B**). In addition, NR2F2 also occupies the genomic loci of two other transcription factors *Egr1* and *Zbtb16* (**Figure S2**) that both manifest reduced mRNA abundance in the *Nr2f2^d/d^* uterus compared with control (**Dataset S1**). The NR2F2 binding events were validated by ChIP-qPCR at 3 and 6 genomic intervals in the *Egr1* and *Zbtb16* loci, respectively (**Figure S2**). These data provide evidence of NR2F2 genome occupancy at loci of *Hand2*, *Egr1*, and *Zbtb16* that are NR2F2 downstream targets, suggesting a direct modulation of the transcription of these major uterine transcription regulators by NR2F2. Notably, PGR occupancy was also detected by ChIP-qPCR in the aforementioned NR2F2-binding regions proximal to the *Hand2* gene body (**Figure S3**), confirming previous observations (NCBI accession number GSE226623) and implicating a potential interaction between NR2F2 and PGR in regulation of *Hand2* expression via recruitment to proximity in the genome.

Next, as a proof of principle, we performed functional assessment on one of the putative enhancers occupied by NR2F2 located at approximately 10-kilobases downstream of the *Hand2* gene (Site 2 in **Figure 5A**). This site was chosen based on the criteria that it has both H3K27ac and H3K4me1 histone marks with NR2F2 occupancy in both uterine ChIP-seq replicates within a 50-kb vicinity of the *Hand2* gene body (**Dataset S3**). The UCSC genome browser liftover tool found correspondence of this mouse putative enhancer in a human genomic region approximately 12-kilobase downstream of the human *HAND2* transcription start site with the genome coordinates chr4:173516002-173519662 in the hg38 genome assembly (**Figure 5C**). The Multiz tool (45) further identified two highly conserved subareas in this genomic region, in which the Remap Atlas (46) shows NR2F2 and PGR occupancy that is documented based on previously published ChIP-seq data (**Figure 5C**). These observations provide evidence to support the subsequent functional assay. For testing the ability of a transcription activator to promote *Hand2* gene expression from this location, two independent guide RNAs (gRNA48 and gRNA49) that target the two conserved subareas were transduced into immortalized human endometrial stromal cells (T-HESC) (47) for the recruitment of the dCas9-VPR CRISPR activator to the target region (**Figure 5C**). qRT-PCR results show an increase of the endogenous *HAND2* mRNA abundance in cells that express gRNA48 and gRNA49 compared with those expressing non-targeting gRNA that serves as a negative control (**Figure 5D**). This finding together with the local NR2F2 occupancy suggest that this enhancer recruits NR2F2 and promotes *HAND2* gene expression.

## Discussion

*In vivo* studies demonstrated both NR2F2 and HAND2 as crucial members of a regulatory network that mediate progesterone dependent epithelial-stromal cross talk to prepare the endometrium ready for embryo implantation (2). NR2F2 has been speculated as an upstream regulator of the *Hand2* gene because progesterone promotes stromal *Hand2* expression (17) and NR2F2 is required for stromal PGR expression (15). Results of the present study provide evidence to show that NR2F2 promotes *Hand2* gene transcription by occupying a nearby region in the non-coding genome of the uterus. The chromatin of this genome interval is modified with enhancer histone marks at early pregnancy and the DNA sequence is conserved between mouse and human. Functional analysis of this genome interval further supports a cis-acting element role of this genome interval that permits the occupying transcription regulators to modulate the *Hand2* gene. These findings place NR2F2 upstream of *Hand2* in the context of natural pregnancy and identify a NR2F2-interacting cis-acting element for transcriptional control of *Hand2*.

In contrast to the defined NR2F2-*Hand2* regulation in the mouse uterus, cultured primary human endometrial stroma cells exhibit a trending decrease of *HAND2* mRNA abundance after silencing NR2F2 (12, 20). The difference between mouse and cell models could be a result of NR2F1 compensation in the human cells. *NR2F1* mRNA is substantially expressed in both primary and immortalized human endometrial stroma cells (20, 48), while NR2F1 expression is below the detection threshold in the mouse endometrium (49). NR2F1 and NR2F2 are structurally similar and functionally interchangeable (49–51). Notably, in the uterus, *Pgr^Cre^* dependent overexpression of human NR2F1 is sufficient to restore the embryo receptivity and the decidualization response (49). These observations suggest a potential functional redundancy between NR2F1 and NR2F2 in human endometrial stroma cells that awaits future investigation.

The putative enhancer 10-kilobase downstream of the mouse *Hand2* gene is occupied by both NR2F2 (**Figure 5A**, site 2) and PGR (NCBI Accession GSE226623). Additionally, PGR ChIP signals are detected in the human endometrial specimen at the secretory phase (52). The PGR occupancy suggests that this site possibly mediates, in part, progesterone’s action on *Hand2* regulation. Moreover, the proximity of NR2F2 and PGR at this site further implicates the potential recruitment of both NR2F2 and PGR into the same transcription regulatory complex by this cis-acting element for the control of *Hand2* expression. NR2F2 is known to physically interact with other nuclear receptors such as the retinoid X receptor (53) and the glucocorticoid receptor (54) as well as with transcription factors including PROX1 (55) and SMAD4 (56). The interaction with other transcription regulators may permit NR2F2 to conduct cell type-specific regulation of gene expression program pertinent to tissue-specific functions, for example, the *Hand2* gene regulation in the uterine stroma. On the same token, the *Hand2* gene’s restricted expression pattern in the stroma and absence in the epithelium could be a result of requiring both NR2F2 (stroma only) and PGR (stroma and epithelia) for expression. This putative uterine *Hand2* enhancer offers a test platform for further assessment of the potential interaction between NR2F2 and PGR on transcription regulation.

While *Nr2f2^d/d^* mice share the phenotype of unopposed estrogen activities in the GD3.5 uterus with *Pgr^AF1/AF1^*, *Sox17^d/d^*, *Errfi1^d/d^*, *Arid1a^d/d^*, and *Sirt1^d/d^* mutants, the underlying mechanisms by which these major regulators manage estrogen responsiveness may vary. Transcriptomic data suggest that *Sirt1* also regulates the HAND2-FGF pathway, which could be achieved through control of NR2F2 expression (40). On the other hand, *Hand2* mRNA abundance is not altered in response to *Arid1a* and *Errfi1* ablation (38, 39) despite an increase of *Fgf18* transcript levels in the *Errfi1^d/d^* uterus compared to the control. These observations together implicate that multiple pathways are required to modulate the estrogen response in the endometrium at early pregnancy under the collective control of these major uterine regulators.

A tightly controlled immune microenvironment is crucial during early pregnancy. For example, uterine mesenchymal cells, including myometrial fibroblasts and decidual stromal cells, express IL33 to support implantation chamber formation and vascular remodeling at early pregnancy (57). However, high maternal serum IL33 levels are associated with miscarriage (58). Similarly, it has been proposed that timely regulated abundance of IL15, another proinflammatory cytokine, in the endometrium is important for recruitment of natural killer cells and trophoblast migration at early pregnancy (20). The elevated levels of *Il33* and *Il15* mRNA in response to *Nr2f2* ablation (**Dataset S1**) suggests that NR2F2 may play a gatekeeper role in coordinating the complex immune responses at the peri-implantation stage via modulating *Il33* and *Il15* expression. Notably, silencing HAND2 in human endometrial stroma fibroblasts also results in an increase of *IL33* and *IL15* mRNA abundance (20). Considering the directional regulation of *Hand2* expression by NR2F2, HAND2 is likely involved in NR2F2 dependent controls of *Il33* and *Il15* expression. In summary, results of the present study support a NR2F2-HAND2 axis in regulation of the uterine immune microenvironment at early pregnancy.

Interestingly, a subpopulation of cells that reside in the subepithelial space of the luminal epithelium retain HAND2 protein immunoreactivities in the absence of NR2F2 (**Figure 3**). Based on the location, it is conceivable to assume the stromal fibroblast origin of these HAND2 expressing cells. One possibility is that signals other than NR2F2 may sustain the HAND2 expression in this subpopulation of stromal cells, while stromal cells distal to the luminal epithelia depend on NR2F2’s regulation of HAND2 expression. Similar to the restricted HAND2 expression, the stromal expression of PGR, a downstream effector of epithelial hedgehog signaling (2), is also limited to the subepithelial space in the *Nr2f2^d/d^* uterus (**Figure 3**). This suggests that stromal NR2F2 is essential to license stromal cells that are located distal to the luminal epithelium to acquire HAND2 and PGR dependent regulatory programs for preparing subsequent embryo implantation. Alternatively, the presence of these HAND2-positive, NR2F2-negative cells could result from a change in cell identity, given that NR2F2 has a role in cell fate determination in progenitor cells (6). NR2F2 loss could also lead to a change in the cell type composition among subpopulations of stromal cells in the endometrium (59–61). Future experimentation to determining the type and the spatial arrangement of composition in the *Nr2f2^d/d^* endometrial cells may help to determine the cellular identity of this subpopulation and shed light on the mechanisms by which NR2F2 controls the homeostasis of various subtypes of stromal cells.

## Data availability statement

All sequencing data are deposited to the Gene Expression Omnibus of the National Center for Biotechnology Information under the accession number GSE232583.

## Ethics statement

Ethical approval was not required for the studies on humans in accordance with the local legislation and institutional requirements because only commercially available established cell lines were used. The animal study was approved by National Institute of Environmental Health Sciences Animal Care and Use Committee. The study was conducted in accordance with the local legislation and institutional requirements.

## Author contributions

YO, FJD, and SPW designed the experiments. YO, EQ, TW, YM-L, SMR, and SPW performed experiments, collected data, and conducted data analyses. JPL provided critical reagent. SPW, FD, and FJD supervised the execution of experiments and data analysis. YO, EQ, TW, YM-L, and SMR wrote parts of the manuscript. SPW and FJD wrote the final draft of the manuscript. All authors contributed to the article and approved the submitted version.

## Supplementary material

Supplementary Figure 1 Gene signature comparison between *Nr2f2* and major uterine regulators by the Illumnia BaseSpace Correlation engine.

Supplementary Figure 2 NR2F2 occupany and enhancer histone mark patterns at the Egr1 and Zbtb16 loci in the GD3.5 uterus and ChIP-qPCR validation on denoted NR2F2 occupying regions. **, *p* < 0.01 compared to the negative control site (NC) presented in by the two-tailed Mann Whitney test. Each group includes datapoints of two biological replicates with technical PCR duplicates. Error bars denote the standard error of the mean.

Supplementary Figure 3 ChIP-qPCR detection of PGR occupany on the NR2F2-binding sites at the *Hand2* locus in the GD3.5 uterus. Genome locations of tested NR2F2-binding sites were denoted in . *, *p* < 0.05 and **, *p* < 0.01 compared to the negative control site (NC) by the two-tailed Mann Whitney test. Each group includes datapoints of two biological replicates with technical PCR duplicates. Error bars denote the standard error of the mean.
