# Supplementary figures and images for: The NR2F2-HAND2 signaling axis regulates progesterone actions in the uterus at early pregnancy
Source: Front Endocrinol (Lausanne). 2023 Aug 18;14:1229033. doi: 10.3389/fendo.2023.1229033 (PMC10473531; doi:10.3389/fendo.2023.1229033)

Figure S1

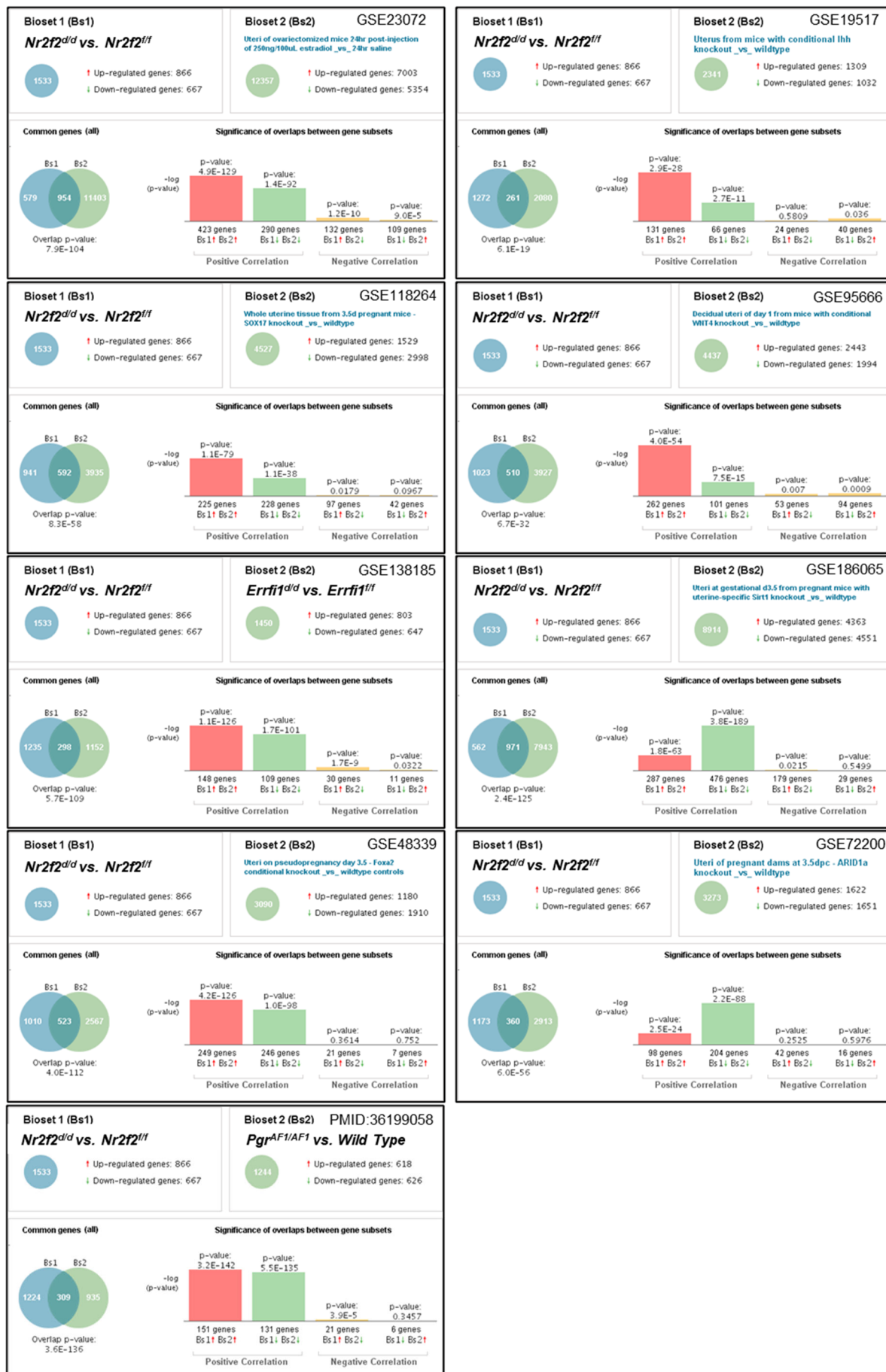

Figure S2

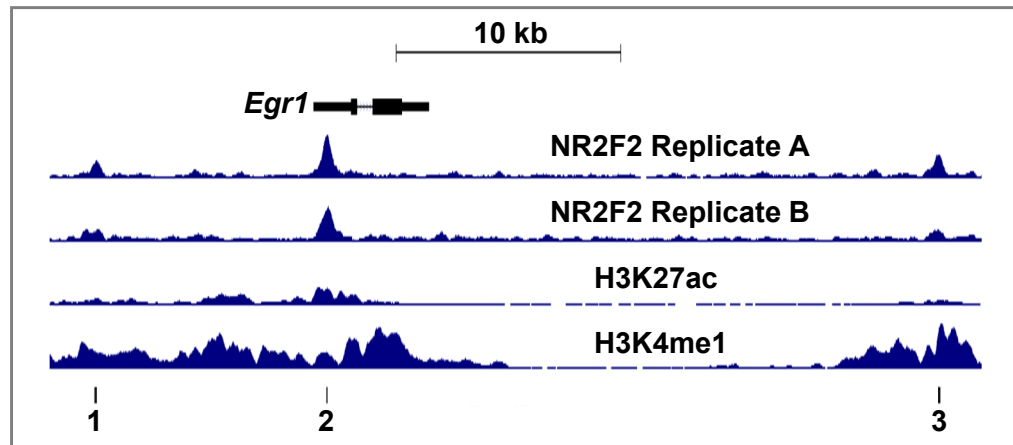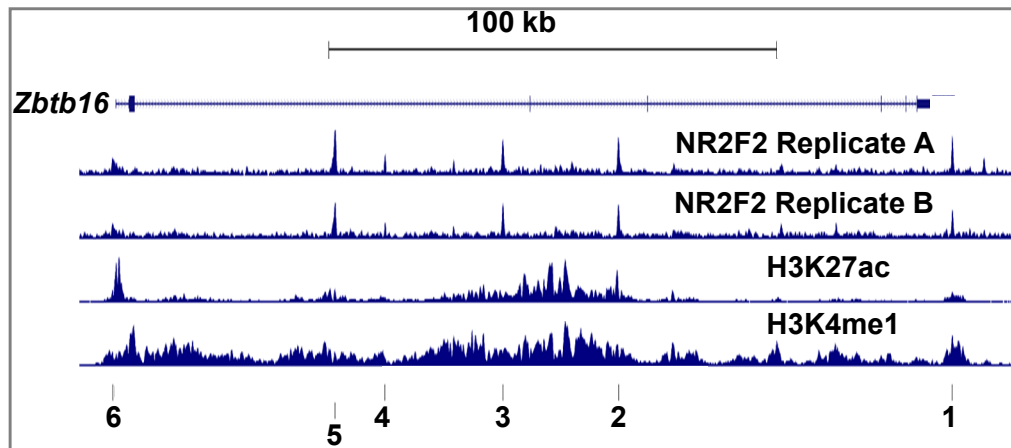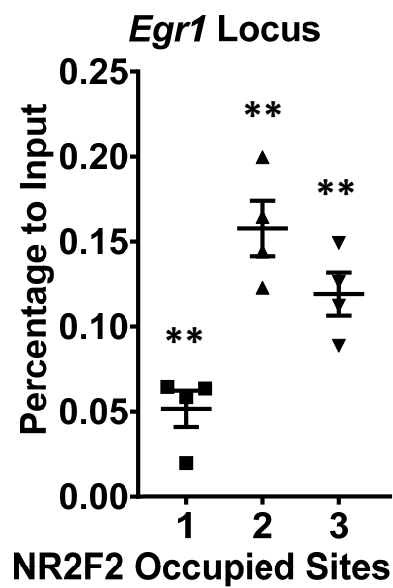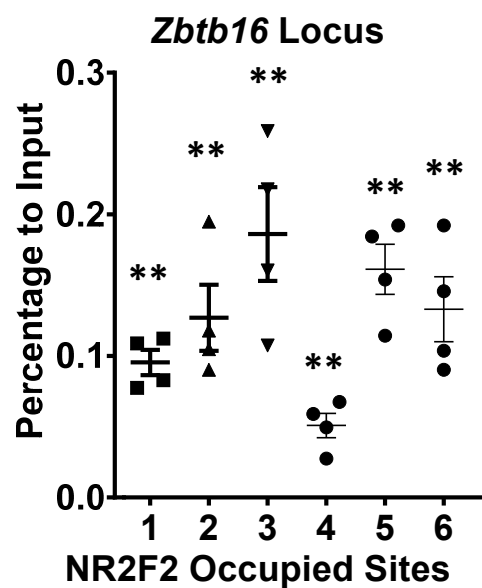

Figure S3

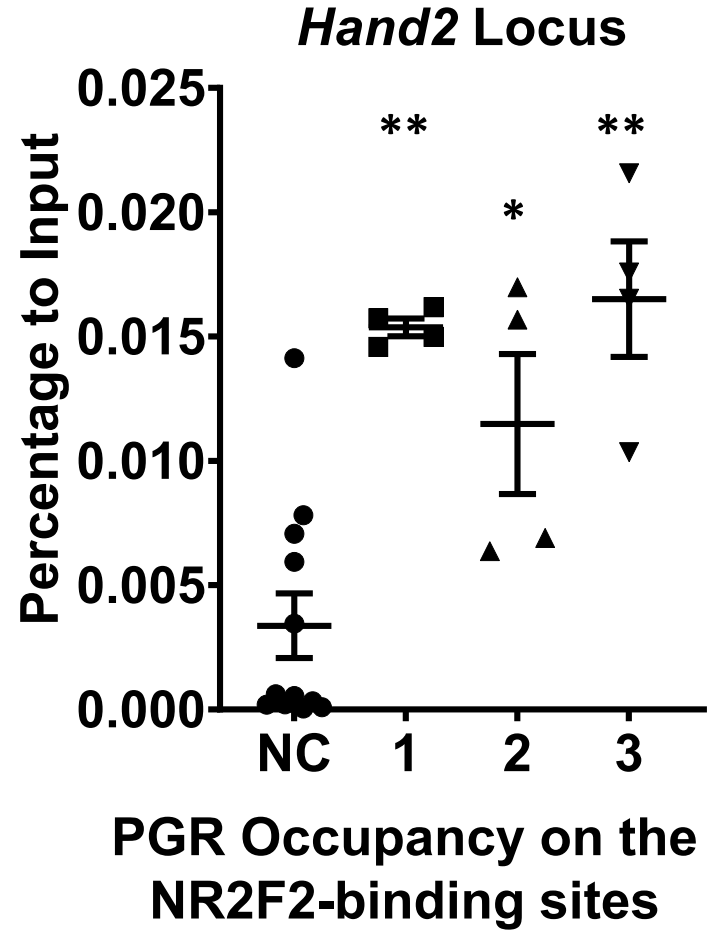

Supplement: Supplementary Figure 1 — Gene signature comparison between Nr2f2 and major uterine regulators by the Illumnia BaseSpace Correlation engine. [file DataSheet_6.pdf]
